# B Cells use Conserved Polarity Cues to Regulate Their Antigen Processing and Presentation Functions

**DOI:** 10.3389/fimmu.2015.00251

**Published:** 2015-05-27

**Authors:** Maria-Isabel Yuseff, Ana Maria Lennon-Duménil

**Affiliations:** ^1^Department of Cellular and Molecular Biology, Pontificia Universidad Católica de Chile, Santiago, Chile; ^2^INSERM-U932, Institut Curie, Paris, France

**Keywords:** B lymphocyte, cell polarity, antigen extraction, processing and presentation, MHC class II, MTOC, lysosomes

## Abstract

The ability of B cells to produce high-affinity antibodies and to establish immunological memory in response to a wide range of pathogenic antigens is an essential part of the adaptive immune response. The initial step that triggers a humoral immune response involves the acquisition of antigens by B cells via their surface immunoglobulin, the B cell receptor (BCR). BCR-engaged antigens are transported into specialized lysosomal compartments where proteolysis and production of MHC class II-peptide complexes occur, a process referred to as antigen processing. Expression of MHC class II complexes at the B cell surface allows them to interact with T cells and to receive their help to become fully activated. In this review, we describe how B cells rely on conserved cell polarity mechanisms to coordinate local proteolytic secretion and mechanical forces at the B cell synapse enabling them to efficiently acquire and present extracellular antigens. We foresee that the mechanisms that dictate B cell activation can be used to tune B cell responses in the context of autoimmune diseases and cancer.

## Introduction

B lymphocytes are tailored to mount antibody responses upon recognition of foreign antigens and display critical roles as antigen-presenting cells that can shape immune responses. The ability of B cells to achieve complete activation and become specialized antibody-secreting cells relies on their capacity to capture external antigens, process and present them as peptide fragments onto MHC class II molecules to CD4+ T cells (1). This interaction, known as T–B cooperation, allows B cells that receive the necessary stimuli required to form germinal centers, to differentiate into high-affinity antibody-producing plasma cells and to develop into memory B cell populations. In this review, we describe how B cells establish immunological synapses with antigen-presenting cells to coordinate the uptake and processing of external antigens. More specifically, we will focus on how proteins that regulate cell polarity impact membrane trafficking and control immune synapse formation, thus enabling B cells to carry out their effector functions.

## Establishment of the B Cell Synapse: A Platform for Signaling and Antigen Extraction

### Activation of B cells by membrane tethered antigens

Within secondary lymphoid organs, B cells encounter soluble antigens smaller than 70 kDa, which can rapidly gain access to lymph nodes through afferent lymph vessels. Such antigens can come in contact with B cells within the follicle by simple diffusion without the need for cell-mediated antigen presentation (2, 3). However, *in vivo* studies based on intra-vital microscopy revealed that larger antigens (with a molecular mass >70 kDa) such as viral aggregates, immune complexes, or antigen-coated particles, which have limited access to the follicle, are able to reach the B cell zone. These antigens are found tethered to the surface of presenting cells, such as macrophages (4, 5), follicular dendritic cells (FDCs) (6), or dendritic cells (7) and are particularly efficient in triggering B cell activation, even at lose densities (8). Indeed, imaging by 2-photon microscopy showed how, within LN follicles, B cells continuously sample and contact antigens that are exposed on the surface of subcapsular sinus macrophages (4). Importantly, interaction of B cells with membrane-bound antigens tethered to the surface of FDCs can also play a role in the selection of high-affinity B cell clones within the germinal centers, where survival signals can be triggered by crosslinking of the B cell receptor (BCR) with immobilized antigens (9). Rapid extraction and processing of these antigens would also allow B cells to interact T helper cells, recently shown to be a crucial factor for affinity-based selection of B cells within germinal centers (10). Altogether, by interacting with antigens presented on the surface of neighboring cells B cells form an immunological synapse that facilitates their efficient extraction and processing.

### Organization of the B cell synapse

**Figure 1:**
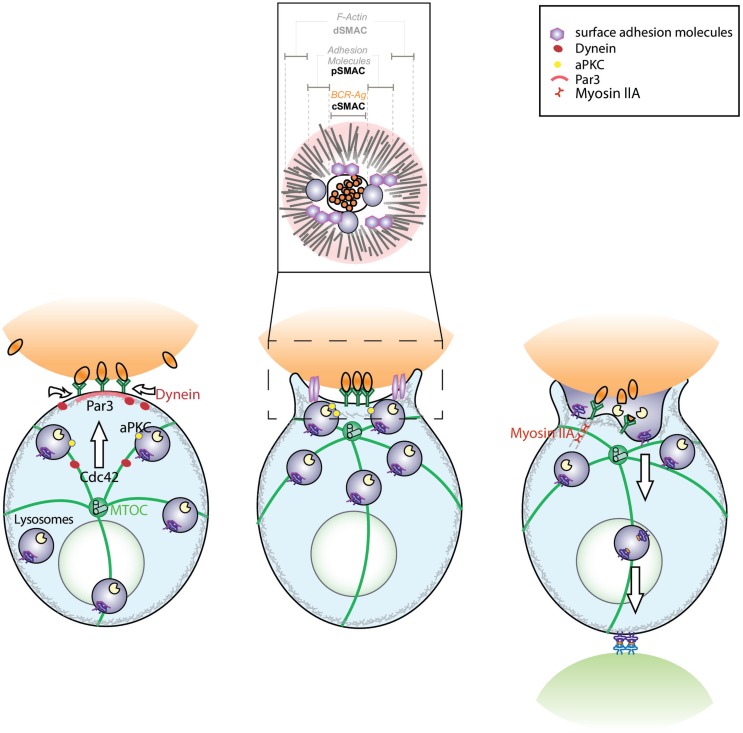
**Model depicting the role of conserved polarity proteins in the extraction and processing of surface-tethered antigen by B cells**. B cell receptor (BCR) engagement by surface-tethered antigen triggers the recruitment of PAR3 to the site of antigen encounter. This leads to the polarization of the microtubule network, where the microtubule-organizing center (MTOC) is translocated toward the immune synapse in a Cdc42-dependent manner. Concomitantly, recruitment of the minus-end molecular motor, Dynein, drives the gathering of antigen–BCR microclusters at the synapse center. Activation of surface adhesion molecules (LFA-1, leukocyte function-associated antigen-1 and VLA-4, very late antigen-4) promote B cell adhesion to the surface, thereby lowering the threshold of activation. These dynamic events lead to the formation of a mature immune synapse characterized by concentric regions: the central supramolecular activation cluster (cSMAC) where BCR engaged with antigen is concentrated, the peripheral SMAC (pSMAC) that contains adhesion molecules such as LFA-1 and the distal SMAC (dSMAC) where actin is enriched (inset). Polarization of the MTOC is also essential for the local recruitment and secretion of MHC II+ lysosomes that promote antigen extraction. This mechanism may act in concert with Myosin IIA-mediated pulling forces that trigger invagination of antigen-containing membranes. Altogether, the B cell immune synapse is essential for B lymphocytes to coordinate efficient antigen extraction with presentation.

The formation of an immunological synapse is initiated upon interaction of the BCR with antigen tethered at the surface of antigen-presenting cells (8). The membrane interface of B cells in contact the antigen undergoes dynamic remodeling, which comprises a rapid actin-dependent membrane spreading response (11, 12) where the antigen–BCR complexes are organized into microclusters that contain signaling molecules, such as Lyn and Syk (13, 14). The spreading reaction exerted by B cells is tightly coupled to their signaling capacity, as cells that recruit fewer signaling molecules to microclusters show deficient spreading responses to membrane-bound antigen (15). Consequently, cell spreading and signaling have a direct impact on the amount of antigen accumulated and extracted at the synapse. Membrane spreading is followed by a contraction phase in which antigen–BCR complexes converge into a central cluster by the concerted action of ezrin–radixin–moesin (ERM) proteins, which link plasma membrane proteins with the actin cytoskeleton (16) and the microtubule-based motor Dynein (17). Ultimately, a highly organized, yet dynamic structure is formed: two concentric regions referred to as the central supramolecular activation cluster (cSMAC), where BCRs are concentrated and the peripheral SMAC (pSMAC) that contains adhesion molecules such as LFA-1 bound to its ligand ICAM-1 (12, 18) (Figure 1, inset). Interestingly, this characteristic arrangement of cell surface receptors was originally observed in CD4+ T cells that establish immune synapses upon recognition of MHC class II-peptide complexes displayed by antigen-presenting cells (18, 19). Accumulation of T cell receptors (TCRs) at the cSMAC is important to control immune receptor signaling as well as their cell surface levels (20). In both T and B cells, engagement of integrins with their respective ligands, on the surface of presenting cells at early stages of antigen recognition was shown to facilitate their activation by promoting adhesion to the target cell and generating co-stimulatory signals (21). Thus, the microenvironment (cell surface receptors and soluble factors) surrounding the tethered antigen can be critical to modulate the outcome of B cell activation (see below). Altogether, the establishment of an immunological synapse is essential for B cells to coordinate efficient receptor signaling and extraction of surface-tethered antigen, two critical components of B cell activation. The impact of polarity proteins in each of these stages shall now be discussed.

### Antigen extraction at the synapse

B cells gather immobilized antigens by coupling cell spreading with BCR–antigen transport to the center of the immune synapse. The molecular basis that accounts for the extraction and uptake of the immobilized antigen is founded on two non-exclusive mechanisms. The first one implicates the local secretion of lysosomes that release proteases and acidify the synaptic cleft where antigen is encountered, allowing its extraction (22). The second one, proposed more recently, suggests that myosin IIA-mediated pulling forces trigger invagination of antigen-containing membranes, which are subsequently internalized into clathrin-coated pits (23). Interestingly, such forces would allow B cells to discriminate between membrane-bound antigens displaying different affinities for the BCR (23). It is likely that both local protease secretion and mechanical forces exerted at the B cell synapse act in concert to promote antigen extraction; however, further studies are required to elucidate the molecular pathways used in each case. The nature of the antigen could determine which mechanism predominates. One could speculate that Myosin II would be more important to acquire antigens with higher affinity, whereas the protease-dependent pathway might be used to extract those of lower affinity unable to be retrieved by myosin IIA-mediated contractions. Additionally, both pathways could be regulated temporarily and exert their functions at particular steps of antigen extraction. Focusing on the spatio-temporal regulation of both protease secretion and Myosin II recruitment at the immune synapse in response to antigens of different affinity should provide useful mechanistic insight.

The presence of co-stimulatory signals, such as the engagement of adhesion molecules during synapse formation, can equally determine how antigens are extracted from the surface of presenting cells. Activation of both B and T cells are fine-tuned by interactions between adhesion molecules expressed on antigen-presenting cells, such as ICAM-1 and LFA-1 (24). In particular, LFA-1 downstream signaling, triggered upon BCR engagement, is critical for efficient B cell activation and spreading upon interaction with immobilized antigens (25). On the other hand, adhesion receptors can sense mechanical forces, through cell–cell contacts, which turn on complex signaling cascades that regulate cytoskeleton organization through Rho GTPases activation (26). Therefore, mechanotransduction pathways can also impact immune synapse organization and function. Indeed, several findings have shown that biophysical properties of antigen-presenting cells, such as membrane stiffness, regulate their ability to activate of T cells (27). Recently, cortical actin cytoskeleton rigidity in DCs was shown to restrict the lateral mobility of adhesion molecules, a state that enhances LFA-1 signaling in T cells during immune synapse formation, thus lowering the threshold for activation (28). Similar studies performed in B cells showed that recruitment of signaling molecules and the formation of BCR–antigen microclusters at the synapse are more efficient when cells encountered antigens tethered to substrates with a higher degree of stiffness (29). Thus, mechanosensing capabilities of B cells also play an important role in the initiation of activation. How physical properties of antigen-presenting cells are transmitted to B cells and whether they have an impact on the mechanisms used for antigen extraction should be addressed.

### Antigen processing by B cells

Antigen recognition and extraction is followed by its internalization into endolysosomal compartments that contain the required processing machinery, such as proteases, MHC class II, and H2-DM molecules, used to cleave antigens and produce peptides for loading on MHC II molecules (30, 31). In contrast to antigens uptaken by fluid phase, which are rapidly degraded in lysosomal compartments, antigen–BCR complexes are transported to non-terminal late endosomes where they can be stored for prolonged periods, thereby enabling B cells to continuously express antigenic peptides on MHCII molecules (32). Additionally, limited proteolysis within these compartments was suggested to produce peptides of a more immunogenic nature (33).

Upon BCR engagement, both the neo-synthesis and trafficking of MHC class II molecules to endocytic compartments (34) are promoted. MHC class II molecules comprise two chains that dimerize shortly after synthesis and are transported from the endoplasmic reticulum (ER) by assembling onto a homotrimeric membrane protein, the invariant chain (Ii). Ii prevents premature association of MHC class II molecules with endogenous peptides and contains in its cytoplasmic tail the targeting signals that deliver MHC class II–Ii complexes from the trans-Golgi network to the endocytic pathway (35). Within endocytic compartments, the luminal domain of Ii is sequentially cleaved by cathepsin proteases, which free the class II-peptide binding groove for loading of the antigenic peptide, a reaction catalyzed by the chaperone H2-DM. A final key step in the process involves the proteolysis of Ii by the cathepsin S, which removes the endosomal retention motif in the cytosolic tail of Ii, thereby allowing MHC class II molecules to be exported to the cell surface (36, 37). The remaining intramembrane portion, corresponding to the N-terminal fragment of Ii, is further cleaved by a signal peptide peptidase-like 2a (SPPL2a) protease, which is essential for its turnover (38). Evidence shows that interfering with proteases of the endocytic pathway of B cells severely impacts both their activation and development. Indeed, B cells from mice lacking SPPL2a accumulate N-terminal fragments of Ii and display altered endocytic membrane trafficking, which causes detects in B cell signaling and maturation (38). Whereas impaired intracellular transport of cathepsins to lysosomes drastically affects the antigen processing and presentation capacity of B cells leading to defective humoral immunity (39).

Altogether, efficient antigen processing relies on the convergence of internalized BCR–antigen complexes with specialized endolysosome compartments. As described here, the antigen processing machinery (MHC class II+ lysosomes) is rapidly recruited to the B cell synapse where internalized antigens are efficiently cleaved to generate MHC class II-peptide complexes that will be presented to CD4+ T cells. The ability of B cells to coordinate vesicle trafficking at the immune synapse relies on conserved polarity proteins and is critical for B cells to become fully competent as antigen-presenting cells.

## B Cell Polarity Regulates B Cell Synapse Formation and Function

### Polarity proteins control membrane trafficking at the B cell synapse

Cell polarity is defined as the asymmetric organization of both functional and structural components and is critical to orchestrate diverse biological functions, ranging from intercellular communications (synapses), directional cell migration to the maintenance of tissue integrity (40). Cell polarity mainly relies on three evolutionarily conserved protein complexes: (1) the Par complex, which comprises Par3 and Par6 and aPKC, (2) the Scribble complex, formed by Scribble, Disks-large (Dlg) and Lethal giant larvae (Lgl), and (3) Crumbs complex (41). Their association with cell surface receptors and elements of the cytoskeleton enable them to organize membrane domains required by cells to carry out specific functions and govern essential biological processes. More recently, cell polarity has emerged as a key element that controls membrane trafficking at the immune synapse of diverse cell types, such as NK cells and cytotoxic T cells (42, 43). These cells rapidly polarize their microtubule-organizing center (MTOC), which guides the delivery of secretory granules toward the site of antigen encounter (44). In a similar fashion, B cells were shown to efficiently recruit MHCII+ lysosomes toward the BCR–antigen interface, by repositioning their MTOC at this site (22). The release of lytic granules by cytotoxic T cells is coupled to centrosome docking at the immune synapse and takes place in subdomians containing lower levels of polymerized actin, suggested to facilitate the local secretion of molecules (44). Whether lysosome secretion at the B cell immune synapse necessary for extracting immobilized antigen is also coordinated with MTOC docking and actin clearance at the synaptic interface remains to be established. Further studies revealed the role of polarity proteins in this process and have thus begun to clarify the picture on how complex membrane trafficking events are controlled at the immune synapse (45). Indeed, previous reports have shown that B cells rely on cell polarity to acquire their antigen presentation function (22, 46). Local lysosome secretion at the site of antigen encounter that promotes extraction of immobilized antigen strictly relies on conserved polarity proteins, the small GTPase Cdc42 and its effector protein aPKC. Silencing of either one of these proteins dramatically affects the capacity of B cells to extract and present immobilized antigen *in vitro* (22). Importantly, genetic evidence also highlights the involvement of theses polarity proteins in B cell functions *in vivo*. aPKC-deficient mice show impaired humoral immune responses *in vivo* (47); whereas conditional Cdc42 knockout mice display important developmental defects in B lymphogenesis (48). The latter study was complemented by a recent analysis performed in mice where Cdc42 was deleted in early B cell progenitors. These mice show defective B cell functions at the level of BCR signaling, presentation of internalized antigen and formation of germinal center responses *in vivo* and are consequently unable to mount humoral responses to antigenic challenges (49).

Recently, the ancestral polarity protein Par3 was found to be enriched at the site of BCR–antigen interaction where it was proposed to act as a landmark of polarity used to guide the further recruitment of key molecules required to form a functional immune synapse (45). Indeed, by promoting MTOC polarization, Par3 was shown to drive the transport and secretion of lysosomes at the synapse of B cells, a critical event in the extraction of immobilized antigen. Interestingly, the localization of Par3 during earlier stages of synapse formation was also shown to be important for the recruitment of the microtubule-based molecular motor, Dynein. Par3 interacts with Dynein to promote directed cell migration in fibroblasts (50). In the case of B cells, the Par3-dependent recruitment of Dynein at the B cell synapse was essential for the centripetal transport of BCR–antigen microclusters to the synapse center (17). Altogether, these data highlight how polarity proteins are implicated in coordinating early and late membrane trafficking events at the immune synapse that are required for antigen processing and presentation to T lymphocytes.

### Role of polarity proteins in membrane trafficking

How polarity proteins influence membrane trafficking in general, and more specifically at the immune synapse, remain largely unknown. In epithelia, interesting links between regulators of endocytic trafficking and conserved polarity machinery have been described where Rab5 and endosomal sorting complex required for transport (ESCRT) regulate apical polarity (51). Conversely, mutations that affect Par3 functions lead to defects in endolysosome trafficking (52). In *Drosophila*, Scrib was shown to regulate retromer-dependent sorting events required for cargo recycling to the cell surface, which is critical to control epithelial organization (53). In T cells, the polarized delivery and fusion of recycling endosomes at the synapse is important to recruit TCRs required for efficient T cell activation (54). Moreover, SNX27, an endosomal sorting protein that interacts with the retromer complex is concentrated at the immune synapse of T cells where it regulates the Ras-EEK signaling pathway (55). Whether this protein interacts with conserved polarity proteins to control polarized membrane trafficking, remains to be elucidated (22). Interestingly, aPKCz was found to be mainly associated to the lysosomes that polarize to the B cell upon BCR engagement (22), where it was required to stabilize their local recruitment at the synapse to promote secretion; however, the associated molecular mechanism remains unknown. Focusing on molecules that regulate the interplay between polarity proteins and the endocytic machinery shall provide new insights on how antigen extraction and processing at the B cell immune synapse are regulated.

Whether polarized membrane trafficking to the B cell synapse involves other intracellular compartments, such as the ER and mitochondria has not been addressed. Indeed, the recruitment of internal membranes could assist membrane spreading as well as provide accessory molecules that locally remodel cortical cytoskeleton and receptor endocytosis, thereby shaping the B cell synapse to acquire its signaling and antigen extraction functions.

## Further Implications for B Cell Polarity in Immune Effector Functions

Does the polarized phenotype induced during B cell synapse formation have an impact on later stages of B cell activation? Early polarization not only guides antigen extraction and presentation but evidence suggests that it can also influence later events, such as migration toward the T cell zone, establishment of germinal centers, as well as asymmetric cell division. Indeed, asymmetric T cell division was shown to result from prolonged synaptic interactions between T cells and antigen-presenting cells. This process was guided by generating a segregation of ancestral polarity proteins, aPKCz and Scribble, giving rise to different T cell progeny with effector or memory fates (56). Asymmetric cell divisions were further described in B cells within germinal centers, where unequal inheritance of fate-associated molecules such as Bcl-6 and the polarity protein aPKC by daughter cells was observed, although the fate of daughter cells arising from this asymmetry remains to be elucidated (57). Interestingly, deficiency in adhesion molecules impaired B cell asymmetric cell division, demonstrating how polarity cues are regulated through the microenvironment. Moreover, asymmetric distribution of antigen within B cells was also observed and shown to be conserved throughout cell division. This property gave rise to daughter cells with unequal antigenic loads, consequently providing them with differential capacities for antigen presentation (58). How polarity proteins impact different stages of B cell activation, such as antigen encounter, migration, or asymmetric cell will most likely give clues on how they achieve their immune functions.

## Conclusion

The current view on how B cells acquire antigens has rapidly evolved from classical receptor-mediated endocytosis toward the formation of a dynamic platform, the immune synapse. This process involves the coordination of complex cellular pathways that regulate local secretion of proteases as well as mechanical forces used for extraction of extracellular antigens. Recent data have exposed a critical role for conserved polarity proteins in orchestrating cytoskeleton remodeling and membrane trafficking at the synaptic interface of B cells. These proteins were shown to regulate local protease secretion at the immune synapse *in vitro*; however, their role in mechanical extraction of antigens remains to be addressed. Nevertheless, given that silencing of polarity proteins in B cells impairs both B cell development and activation *in vivo* it has become apparent that they are essential for proper humoral responses. Therefore, identifying new proteins that bridge cell polarity to membrane trafficking and coordination of mechanical forces shall allow a better understanding on how B cells acquire their antigen-presenting functions. Elucidating these molecular pathways can also provide valuable therapeutical targets for immunomodulation, in particular, in the context of autoimmune diseases and cancer.

## Conflict of Interest Statement

The authors declare that the research was conducted in the absence of any commercial or financial relationships that could be construed as a potential conflict of interest.
